# Mesoporous Nickel Oxide (NiO) Nanopetals for Ultrasensitive Glucose Sensing

**DOI:** 10.1186/s11671-018-2435-3

**Published:** 2018-01-11

**Authors:** Suryakant Mishra, Priyanka Yogi, P. R. Sagdeo, Rajesh Kumar

**Affiliations:** Material Research Laboratory, Discipline of Physics & MEMS, Indian Institute of Technology Indore, Simrol, Indore, 453552 India

**Keywords:** NiO nanopetals, Electrochemical Sensing, Glucose

## Abstract

Glucose sensing properties of mesoporous well-aligned, dense nickel oxide (NiO) nanostructures (NSs) in nanopetals (NPs) shape grown hydrothermally on the FTO-coated glass substrate has been demonstrated. The structural study based investigations of NiO-NPs has been carried out by X-ray diffraction (XRD), electron and atomic force microscopies, energy dispersive X-ray (EDX), and X-ray photospectroscopy (XPS). Brunauer–Emmett–Teller (BET) measurements, employed for surface analysis, suggest NiO’s suitability for surface activity based glucose sensing applications. The glucose sensor, which immobilized glucose on NiO-NPs@FTO electrode, shows detection of wide range of glucose concentrations with good linearity and high sensitivity of 3.9 μA/μM/cm^2^ at 0.5 V operating potential. Detection limit of as low as 1 μΜ and a fast response time of less than 1 s was observed. The glucose sensor electrode possesses good anti-interference ability, stability, repeatability & reproducibility and shows inert behavior toward ascorbic acid (AA), uric acid (UA) and dopamine acid (DA) making it a perfect non-enzymatic glucose sensor.

**Electronic supplementary material:**

The online version of this article (10.1186/s11671-018-2435-3) contains supplementary material, which is available to authorized users.

## Background

Diabetes, a chronic disease in which glucose level increases in blood and if undiagnosed and untreated, can be very hazardous for health and eventually may lead to death [1, 2]. Different therapy regimes in the management of diabetes include drugs’ dose adjustment according to the level of glucose in the blood as a result of compromised insulin level, main cause of the disease. Hence, accurate and reliable glucose sensor to sense the level in the blood is the most important parameter in managing diabetes. Generally, glucose sensor works on the use of an enzyme, glucose oxidase (GOx), which converts glucose into gluconic acid and H_2_O_2_ [3–7]. The concentration of glucose is determined by monitoring the number of electrons flowing through electrode for the formation of hydrogen in the form of peroxide [8]. In enzymatic biosensors, quantitative sensing is done by controlling the potential and measuring the current as a result of substance (to be sensed) reacting with the active area of the material (acting as sensor) on the working electrode. Enzymatic glucose sensors, working on the same principle, display high sensitivity to glucose. Limitations with these sensors include their shorter life span, the environmental conditions such as temperature, pH value, and toxicity of the chemical used. To address these issues, many metal oxide-based non-enzymatic glucose sensors have been developed in recent time [9–14]. The sensing mechanism of these non-enzymatic glucose sensors is based on oxidation of glucose, by metal-oxide ion near the surface of the electrode, to gluconolactone. In electrochemical sensing, cyclic voltammetry (CV) proves to be an efficient technique due to its high sensitivity at low detection limits, accurate quantitative analysis, and fast and clear characterization [15, 16]. These oxide-based glucose sensors certainly have potential to be used in real diagnosis and need further study.

There are increasing interests on fabrication of electrodes with low-cost metal-oxide materials, such as NiO, CuO, TiO_2_, ZnO, and composites which can show high sensitivity toward glucose by improving electro-catalytic activity [17–24]. When it comes to reaction-based sensing, nanomaterials could be of interest as they can provide more surface area for reaction and hence better sensing. In recent times, a variety of materials in nanostructured form have shown great potential in sensing, electronics, and optoelectronics [25–27]. Established fact about nanostructures is the capability of tailoring a physical property by changing its size and/or morphology which gives the versatility to the nanomaterials to be used in diverse applications. Hence, for sensors also design of electrodes surface is one of the key parameters. Amongst plenty, Ni-based nanomaterials exhibit remarkable properties, such as catalysis [28–30] and high sensitivity due to large surface-to-volume ratio. An economic yet sensitive glucose sensor can be a reality with NiO nanostructure-based sensors by appropriately designing the device and synthesizing the material. In this paper, a working electrode consisting of petal-like NiO nanostructures for glucose sensing via electrochemical study has been fabricated to be used as the active compound. Fluorene-doped tin oxide (FTO)-coated conducting glass substrate has been used to grow the NiO nanostructures (NSs) by hydrothermal technique.

## Experimental

Nickel nitrate precursor mixed with potassium persulfate in the presence of less amount of ammonium solution has been used for the alignment during the preparation of these NiO NSs. After 5 h of continuous heating at 150 °C, deposited film was rinsed with deionized water and dried in air. Subsequently, the NiO-NSs film was annealed at 250 °C for 2 h. Uniform and well-aligned NiO NSs were obtained on the conducting surface of FTO-coated glass. The microstructure of the film was investigated by a XRD (Rigaku SmartLab X-ray diffractometer using monochromatic Cu-Kα radiation *λ* = 1.54 Å) along with electron microscopy (Supra55 Zeiss). Energy dispersive X-ray spectroscopy (Oxford Instrument) and X-ray photoelectron spectrometer (ESCA System, SPECS GmbH, Germany) with Al Kα radiation (1486.6 eV) have been used for the elemental confirmation. Atomic force microscopy has been performed on a Bruker (MultiMode 8-HR) machine, and analysis of high-resolution nanostructures were carried out using WSxM software [31]. For glucose sensing with NiO-NSs, appropriate electrochemical measurements have been performed using Keithley 2450-EC electrochemical work station. Brunauer–Emmett–Teller (BET) method was also employed on Autosorb iQ, version 1.11 (Quantachrome Instruments) for surface analysis.

## Results and Discussion

**Fig. 1:**
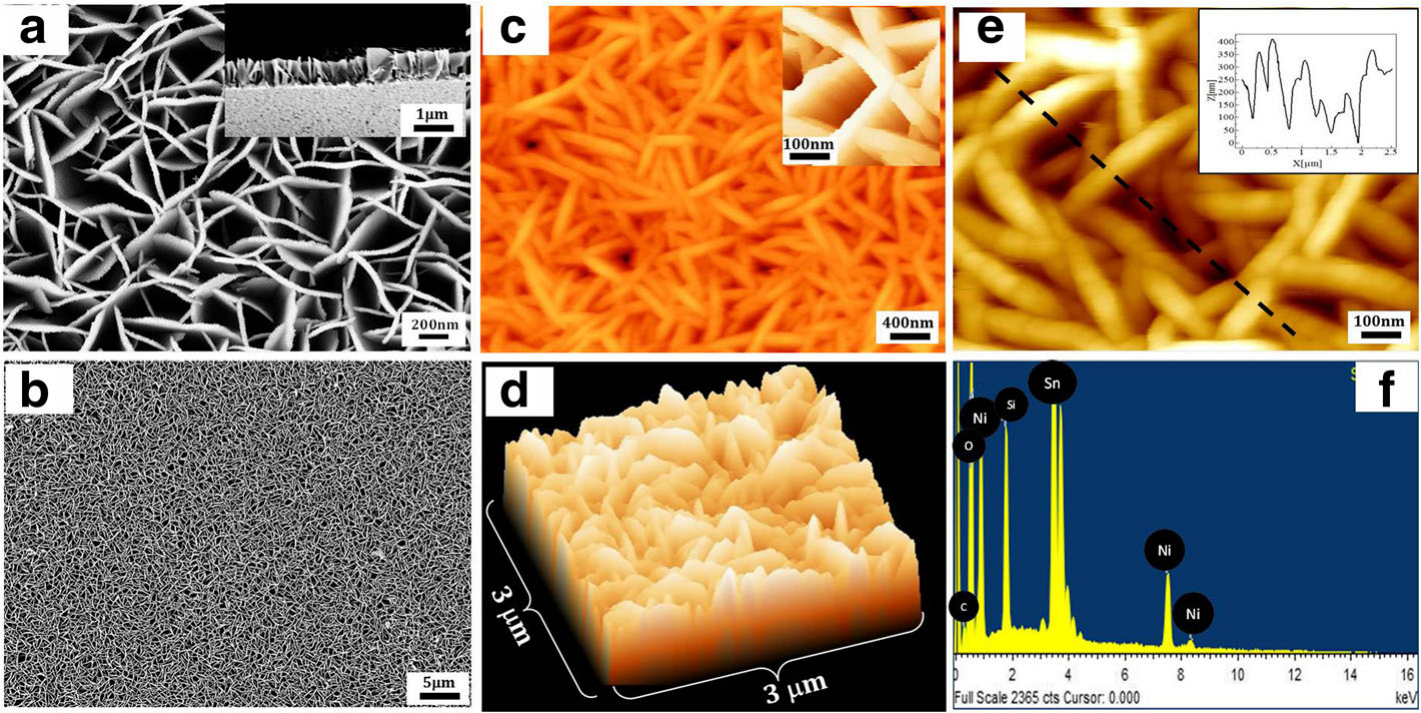
**a**, **b** Surface morphologies of NiO nanostructures showing petal-like structure with its cross-sectional view (inset). **c**–**e** AFM images with line profiling. **f** EDX spectra for elemental conformation

Microstructural details and morphology of NiO NSs have been studied using electron microscopy and atomic force microscopy (AFM). Figure 1a shows very dense rose-petal-like structures grown on the FTO-coated conducting glass substrate. Thickness of these petals is approximately 25–30 nm covered with very fine thorns like structures on the top of it. The film is dense and uniform over more than hundred microns. The uniformity over larger areas makes it eligible for sensing applications. Cross-sectional view of the NiO NSs can be seen in inset of Fig. 1a which shows vertical alignment and the height of the petals. TEM micrograph of these NiO NSs can be seen in Additional file 1: Figure S1. Figure 1b shows the SEM image of NiO nanopetals showing that uniform NiO NPs are grown over wide area. More details about shape and sizes of these nanopetals have studied using AFM images in Fig. 1c–e. Figure 1c, d shows two- and three-dimensional AFM images, respectively. It shows approximately uniformly distributed petals with highly dense nanopetals (NPs) aligned vertically. AFM images in Fig. 1e and inset of Fig. 1c show NiO NSs at higher resolution. Black line on Fig. 1e shows line profiling of the nanostructure, which gives information about the average thickness of the NPs. It is apparent that nanopetals have widths in the range of ~ 25-30 nm. Energy dispersive X-ray (EDX) spectrum in Fig. 1f shows chemical composition of NiO NPs suggestive of high purity NiO NSs with adequate Ni/O ratio. Some peaks corresponding to elemental Tin (Sn) can also be seen from FTO-coated glass used as substrate. Figure 1, clearly demonstrate that dense NiO NSs in the petal-shape have been fabricated uniformly, with some porosity, on an FTO coated glass substrate.

**Fig. 2:**
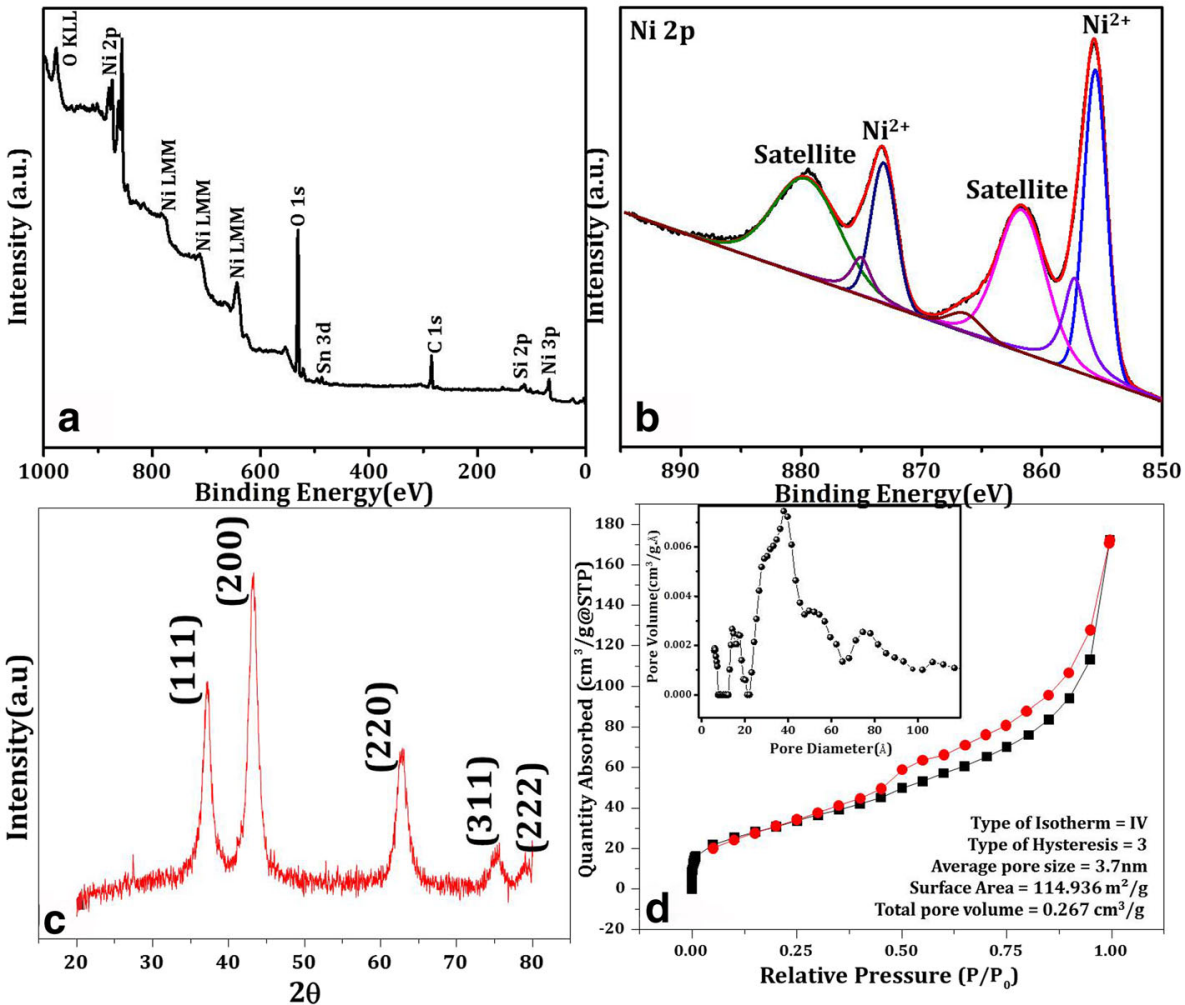
Constituent analysis of the fabricated NiO nanopetals using XPS **a** survey scan, **b** deep scan of 2p Ni, **c** XRD for the structural analysis, and **d** surface area and textual study using BET isotherm measurement by N_2_ adsorption/desorption

X-ray photoelectron spectroscopy (XPS) is performed for the analysis of constituents and surface chemical compositions of NiO nanopetals. The XPS survey scan (Fig. 2a) depicts composition of nickel and oxygen with the substrate peak of tin (Sn) which is consistent with the EDX results. Two characteristic Ni 2p peaks are observed at about 855.7 eV (2p_3/2_) and 873.4 eV (2p_1/2_) in high resolution scan (Fig. 2b). The deconvoluted spectrum contains seven peaks with two stronger peaks at 855.7 and 873.4 eV correspond to Ni^2+^ in Ni–O bonds, with two satellite (weak) peaks [32]. XRD pattern in the Fig. 2c clearly shows diffraction peaks, in the order of decreasing XRD peak intensities, at 43°, 37°, 63°, 76°, and 79°, respectively. The peak positions and their relative intensities are in good agreement with the face centered cubic (FCC) structure of NiO-NSs revealing a crystalline nature of the NPs [33]. Above–mentioned morphological and structural characterization of prepared substrate predicts the presence of low dimensional petal like structures of NiO and the same will be investigated for possible glucose sensing properties.

As mentioned earlier, basis of the sensing mechanism is the reactivity of glucose with NiO thus needing higher surface areas, which should be analyzed before investigating the sensing properties. The specific surface area and other parameters, like type of isotherm, average pore size, and total pore volume have been obtain by the *N*_2_ adsorption/desorption using BET method. Figure 2d reveals type IV isotherm and type-H3 hysteresis when measured at 77 K with the relative pressure range of 0.025 ≤ *P*/*P*_0_ ≤ 1.00 [18]. The measured surface area, estimated by BET and Langmuir methods in the *P*/*P*_*0*_ range of 0.05–0.30, is found to be 114.936 m^2^/g and pore size distribution around 3.7 nm. This indicates NiO NPs are mesoporous with relatively uniform pore size distribution. The total pore volume in the sample is found to be 0.267 cm^3^/g as estimated at a relative pressure (*P*/*P*_0_) of 0.99.

**Fig. 3:**
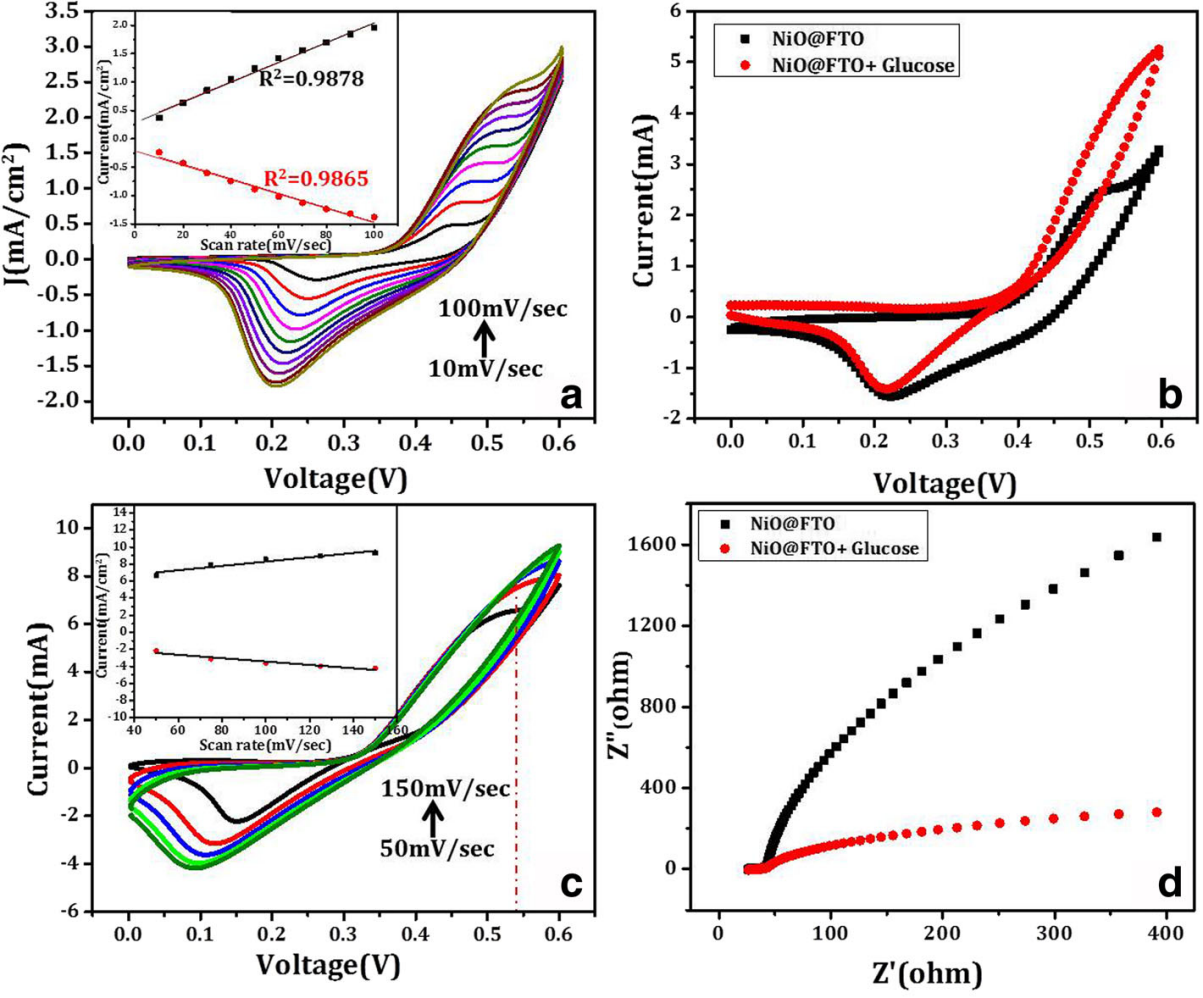
**a** Cyclic voltammetry (CV) of NiO-NPs@FTO on various scan rates. **b** Elctrochemical glucose(10 μM) sensing using CV technique. **c** CV scan of glucose immobilized NiO-NPs@FTO electrode at various scan rates. **b** Electrochemical impedance spectroscopy (EIS) to show glucose sensing. Insets in **a** and **c** show a linear variation of current as a function of scan rate

An adequate surface appears available for glucose sensing of the NiO-NPs has been studied below using electrochemical CV measurements as shown in Fig. 3. For CV measurements, a three-electrode system has been employed with NiO-NPs@FTO sample as working electrode, Ag/AgCl (1 M KCl) and platinum wire used as reference and counter electrodes, respectively. Figure 3a shows *I*–*V* curves with different voltage sweep rates varying between 10 and 100 mV/s. The electrode is very stable as tested by repeating the CV scans for 3000 cycles (Additional file 1: Figure S2). It is evident from Fig. 3a that a current of ~ 0.25 mA/cm^2^ was flowing at a scan rate of 10 mV/s (black curve) and increases to ~ 2.5 mA/cm^2^ when scan rate was increased to 100 mV/s (light green curve). A ten times current increase by increasing the scan rate by ten times means a linear variation between the two. Such a linear variation in current as a function of a scan rate, as evident in Fig. 3a inset, is most often assigned to be originating due to a surface-controlled reaction and will be better for sensing applications.

For sensing study, CV measurements have been carried out with NiO NSs film as working electrode (NiO-NPs@FTO) at a scan rate of 50 mV/s with (red) and without (black) glucose (5 mM), in the presence of 0.1 M NaOH electrolyte as shown in Fig. 3b. The CV plots recorded at different scan rates in the presence of glucose have also been shown in Fig. 3c which also shows increased current values as compared to non-glucose case and further increases with increasing scan rates. This scan rate-dependent CV curves in Fig. 3c are consistent with the discussions above pertaining to the glucose sensing and surface controlled reaction. As can be seen from the black and red curves in Fig. 3b, a reaction peak current is observed, indicating that NiO-NPs@FTO electrode undergoes the redox reaction in the potential range of 0.0 to 0.6 V. The peak current value gets doubled in the presence of glucose, i.e., the current of NiO-NPs@FTO electrode with glucose is larger than the one without glucose which can be attributed to oxidation of glucose molecule immobilized within larger surface area of the NiO NSs. This appears to be the most likely mechanism of glucose sensing as can be supported by the following redox reactions taking place at appropriate sites.

1 $$ \mathrm{NiO}+{\mathrm{H}}_2\mathrm{O}\to \mathrm{NiO}\mathrm{OH} $$

2 $$ \mathrm{NiO}\mathrm{OH}+\mathrm{glucose}\to \mathrm{NiO}+{\mathrm{H}}_2{\mathrm{O}}_2+\mathrm{gluconolactone} $$

3 $$ \mathrm{Gluconolactone}\to \mathrm{gluconic}\  \mathrm{acid} $$

4 $$ \mathrm{Gluconic}\  \mathrm{acid}+{\mathrm{H}}_2\mathrm{O}\to {\mathrm{gluconate}}^{\hbox{-} }+{\mathrm{H}}^{+} $$

During CV measurement, Ni^2+^ oxidizes into Ni^3+^ by aqueous electrolytic solution present in the cell at NiO-NPs@FTO electrode (reaction 1). Oxidized Ni^3+^ works as catalyst for glucose and oxidizes glucose by reducing itself (reaction 2). On oxidation, glucose converts to gluconolactone which consequently gets converted immediately to gluconic acid (reaction 3) and this compound reacts with water molecules to form gluconate and hydronium ions (reaction 4). These ions near the surface of working electrode result in increased current as detectable signal with a very good specific sensitivity of 3.9 μA/μM/cm^2^.

In order to further support the “glucose-doping” induced enhancement in electric conductivity, electrochemical impedance spectroscopy (EIS) of NiO NP-fabricated working electrode has been measured with and without glucose (Fig. 3d). A single depressed semicircle in the high-frequency region and an inclined line in the low frequency region can be seen in the Nyquist (cole-cole) plot in Fig. 3d. Generally, the high-frequency semicircle shows the electrochemical reaction impedance between the glucose present in the electrolytic solution and NiO nanostructure interface, whereas inclined line in the lower frequency region shows the active material (NiO) and conducting electrode interface impedance [34]. Effect of glucose on the cole-cole plot in Fig. 3d is clearly distinguishable, and thus, the same measurement can be utilized to sense the presence of glucose. This clearly exhibits the glucose sensing property of the material which is nanopetal shaped NiO NSs.

**Fig. 4:**
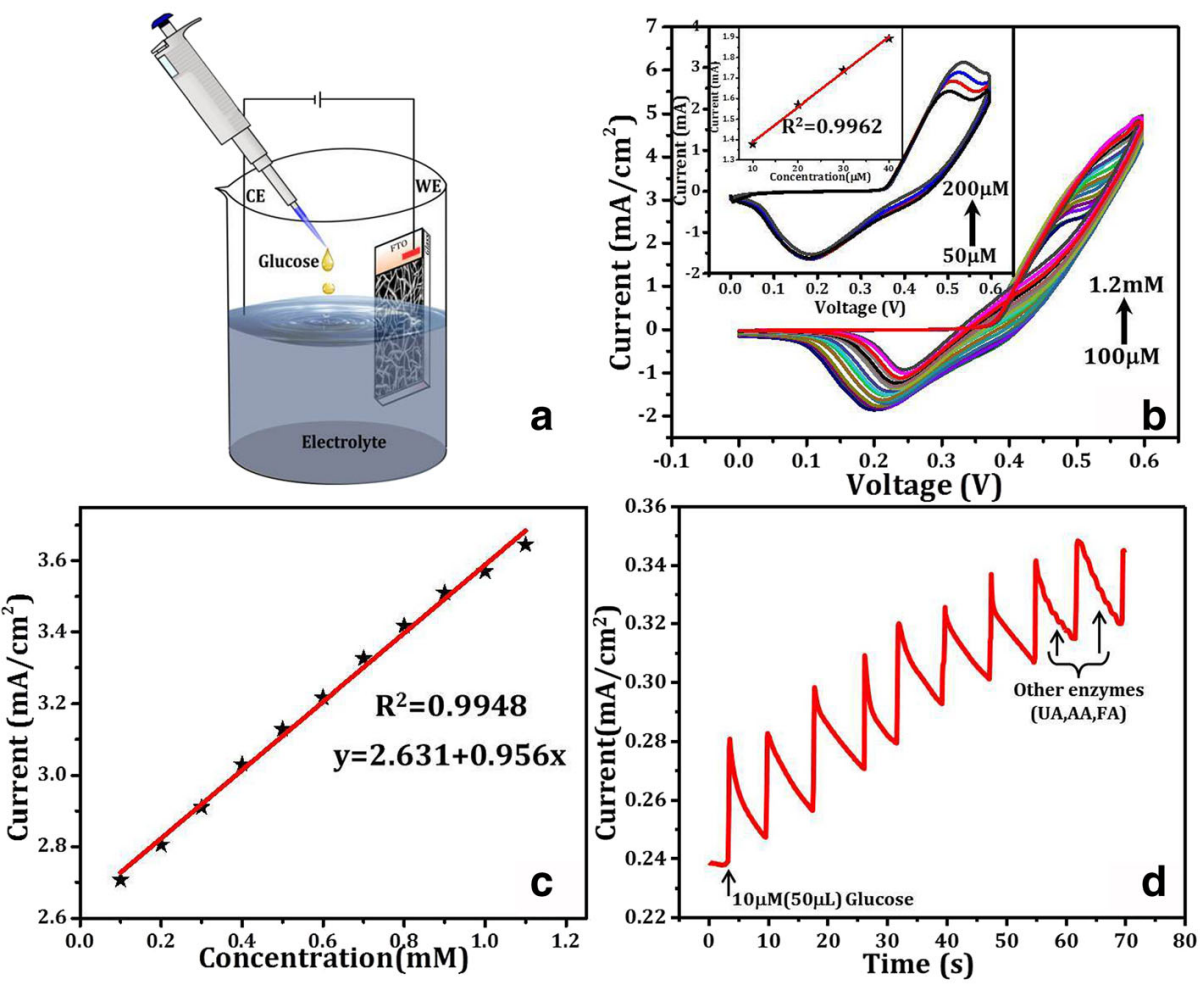
**a** Schematic illustration of electrochemical glucose sensing setup using NiO-NPs@FTO as working electrode with supporting electrolyte NaOH (0.1 M). **b** Sequential glucose addition of 50 μM during CV scan with its magnifying view in the inset. **c** Linear relation of glucose concentration with current **d** amperometric response (at + 0.5 V) on a 10-μM glucose addition

The repeatability of a device is one of the important parameters for effective performance as real sensor. Figure 4a is the electrochemical cell for the glucose sensing using CV and amperometric techniques. Figure 4b corresponds to CV scan of NiO-NPs@FTO in the presence of various glucose concentrations from 100 μM–1.2 mM. Figure 4c shows linear relation of glucose concentration with current density having a linear fitting factor (*R*^2^) of 0.9948. Figure 4d shows amperometric behavior of NiO-NPs@FTO electrode on addition of aqueous glucose solution of different amounts in 0.1 M NaOH electrolyte as sensed at + 0.5 V. At this bias, the NiO-NPs@FTO electrode exhibits systematic changes in the current when 50 μL glucose solution of concentration, 1 μM is added in the electrolyte. Further, to illustrate the exclusive glucose sensing behavior, effect of other compounds present with glucose-like uric acid (UA), ascorbic acid (AA), and folic acid (FA) was checked by carrying out control experiments. Responses of the mentioned species at various concentrations were studied by adding these enzymes at 57th and 65th seconds (arrow marked in Fig. 4d) which do not show any significant changes in the current during amperometric measurement whereas glucose was sensed when added in between at 60th second. Selectivity of glucose sensing in comparison with other compounds can be seen more clearly in Additional file 1: Figure S3. Another important observation is the reduction in current after glucose induced spike, which makes the sensor reusable. The NiO NS electrode shows very good sensitivity as compared to various other sensor electrodes as can be seen in Table 1 which summarizes some of the recent glucose sensing electrodes. A superior sensitivity of the NiO NS-based electrode (bottom row in Table 1) makes it a good candidate for glucose sensing applications on which further studies can be done on real samples like blood or foods as applicable.

**Table 1:** Comparative study of analytical performance of the NiO-NPs@FTO-fabricated glucose sensor

Type of electrode	Sensitivity (μA/μM/cm^2^)	Detection potential (V)	Reference
Ti/TiO2 nanotube arrays/Ni	0.20	055	Wang et al. [10]
Ni nano-sphere/RGO	0.15	0.46	Yang et al. [35]
Ni nanoparticles loaded MWCNT	1.44	0.4	Nie et al. [36]
Ni nanoparticles loaded carbon nanofibers	0.42	0.6	Liu et al. [37]
3D porous Ni nano-network	2.90	0.5	Niu et al. [38]
NiO-NPs@FTO	3.90	0.5	In this work

## Conclusions

In summary, an excellent glucose sensing behavior with improved sensitivity has been achieved by using an electrode with hydrothermally grown highly dense, aligned NiO nanostructures (NSs), with high surface to volume ratio. The NiO NSs, grown by the simple technique, show better glucose sensing capabilities in terms of stability and sensitivity as compared to its counterparts grown by some others technique. The proposed sensor electrode demonstrates wide range of detection of glucose concentrations with high-specific sensitivity of 3.9 μA/μM/cm^2^ and a fast response time of less than 1 s. In addition to this, it shows inert response to the other enzymes present with glucose like ascorbic acid, folic acid, and uric acid, which makes it efficient non-enzymatic glucose sensor. All these obtained results indicate that the proposed glucose sensor can be an efficient analytical tool for the monitoring of glucose concentrations in drugs, human serum, and can be used in biomedical-related applications.

## Additional file

Additional file 1: Supporting information. (PDF 521 kb)

## References

[1] Wong TY, Cheung CMG, Larsen M (2016). Diabetic retinopathy. Nat Rev Dis Primer.

[2] (2015) Diabetic kidney disease. Nat Rev Dis Primer 1:15038. 10.1038/nrdp.2015.38 10.1038/nrdp.2015.38 27228171

[3] Lee H, Choi TK, Lee YB (2016). A graphene-based electrochemical device with thermoresponsive microneedles for diabetes monitoring and therapy. Nat Nanotechnol.

[4] Xiang Y, Lu Y (2011). Using personal glucose meters and functional DNA sensors to quantify a variety of analytical targets. Nat Chem.

[5] Bai J, Zhou B (2014). Titanium dioxide nanomaterials for sensor applications. Chem Rev.

[6] Heller A, Feldman B (2008). Electrochemical glucose sensors and their applications in diabetes management. Chem Rev.

[7] Chandran GT, Li X, Ogata A, Penner RM (2017). Electrically transduced sensors based on nanomaterials (2012–2016). Anal Chem.

[8] Wang J (2008). Electrochemical glucose biosensors. Chem Rev.

[9] Lin Y, Lu F, Tu Y, Ren Z (2004). Glucose biosensors based on carbon nanotube nanoelectrode ensembles. Nano Lett.

[10] Wang C, Yin L, Zhang L, Gao R (2010). Ti/TiO2 nanotube array/Ni composite electrodes for nonenzymatic amperometric glucose sensing. J Phys Chem C.

[11] Xu J, Xu N, Zhang X (2017). In situ fabrication of Ni nanoparticles on N-doped TiO2 nanowire arrays by nitridation of NiTiO3 for highly sensitive and enzyme-free glucose sensing. J Mater Chem B.

[12] Xu J, Xu N, Zhang X (2017). Phase separation induced rhizobia-like Ni nanoparticles and TiO2 nanowires composite arrays for enzyme-free glucose sensor. Sens Actuators B Chem.

[13] Başkaya G, Yıldız Y, Savk A (2017). Rapid, sensitive, and reusable detection of glucose by highly monodisperse nickel nanoparticles decorated functionalized multi-walled carbon nanotubes. Biosens Bioelectron.

[14] Qian Q, Hu Q, Li L (2018). Sensitive fiber microelectrode made of nickel hydroxide nanosheets embedded in highly-aligned carbon nanotube scaffold for nonenzymatic glucose determination. Sens Actuators B Chem.

[15] Wang J, Xu L, Lu Y (2016). Engineered IrO2@NiO core–shell nanowires for sensitive non-enzymatic detection of trace glucose in saliva. Anal Chem.

[16] Saraf M, Natarajan K, Mobin SM (2016). Non-enzymatic amperometric sensing of glucose by employing sucrose templated microspheres of copper oxide (CuO). Dalton Trans.

[17] Si P, Ding S, Yuan J (2011). Hierarchically structured one-dimensional TiO2 for protein immobilization, direct electrochemistry, and mediator-free glucose sensing. ACS Nano.

[18] Tian H, Jia M, Zhang M, Hu J (2013). Nonenzymatic glucose sensor based on nickel ion implanted-modified indium tin oxide electrode. Electrochim Acta.

[19] Ibupoto ZH, Khun K, Lu J, Willander M (2013). The synthesis of CuO nanoleaves, structural characterization, and their glucose sensing application. Appl Phys Lett.

[20] Wang JX, Suna XW, Wei A (2006). Zinc oxide nanocomb biosensor for glucose detection. Appl Phys Lett.

[21] Tee SY, Ye E, Pan PH (2015). Fabrication of bimetallic Cu/Au nanotubes and their sensitive, selective, reproducible and reusable electrochemical sensing of glucose. Nano.

[22] Mu Y, Jia D, He Y (2011). Nano nickel oxide modified non-enzymatic glucose sensors with enhanced sensitivity through an electrochemical process strategy at high potential. Biosens Bioelectron.

[23] Liu X, Yang W, Chen L, Jia J (2017). Three-dimensional copper foam supported CuO nanowire arrays: an efficient non-enzymatic glucose sensor. Electrochim Acta.

[24] Tee SY, Teng CP, Ye E (2017). Metal nanostructures for non-enzymatic glucose sensing. Mater Sci Eng C.

[25] Mishra S, Pandey H, Yogi P (2017). Interfacial redox centers as origin of color switching in organic electrochromic device. Opt Mater.

[26] Mishra S, Yogi P, Saxena SK (2016). Fano scattering: manifestation of acoustic phonons at the nanoscale. J Phys Chem Lett.

[27] Chan JYT, Ang SY, Ye EY (2015). Heterogeneous photo-Fenton reaction on hematite (α-Fe2O3){104}, {113} and {001} surface facets. Phys Chem Chem Phys.

[28] Kwong WL, Lee CC, Messinger J (2017). Scalable two-step synthesis of nickel–iron phosphide electrodes for stable and efficient electrocatalytic hydrogen evolution. J Phys Chem C.

[29] Popczun EJ, McKone JR, Read CG (2013). Nanostructured nickel phosphide as an electrocatalyst for the hydrogen evolution reaction. J Am Chem Soc.

[30] Nail BA, Fields JM, Zhao J (2015). Nickel oxide particles catalyze photochemical hydrogen evolution from water—nanoscaling promotes P-type character and minority carrier extraction. ACS Nano.

[31] Horcas I, Fernández R (2007). WSXM: a software for scanning probe microscopy and a tool for nanotechnology. Rev Sci Instrum.

[32] Chen Y, Wang Y, Sun P (2015). Nickel oxide nanoflake-based bifunctional glass electrodes with superior cyclic stability for energy storage and electrochromic applications. J Mater Chem A.

[33] Mishra S, Yogi P, Saxena SK, et al (2017) Significant field emission enhancement in ultrathin nano-thorn covered NiO nano-petals. J Mater Chem C. 10.1039/C7TC01949A

[34] Méndez PF, López JR, López-García U (2017). Voltammetric and electrochemical impedance spectroscopy study of Prussian blue/polyamidoamine dendrimer films on optically transparent electrodes. J Electrochem Soc.

[35] Yang D, Liu P, Gao Y (2012). Synthesis, characterization, and electrochemical performances of core-shell Ni(SO4)0.3(OH)1.4/C and NiO/C nanobelts. J Mater Chem.

[36] Nie H, Yao Z, Zhou X (2011). Nonenzymatic electrochemical detection of glucose using well-distributed nickel nanoparticles on straight multi-walled carbon nanotubes. Biosens Bioelectron.

[37] Liu Y, Teng H, Hou H, You T (2009). Nonenzymatic glucose sensor based on renewable electrospun Ni nanoparticle-loaded carbon nanofiber paste electrode. Biosens Bioelectron.

[38] Niu X, Lan M, Zhao H, Chen C (2013). Highly sensitive and selective nonenzymatic detection of glucose using three-dimensional porous nickel nanostructures. Anal Chem.

