# Supplementary material for: Mesoporous Nickel Oxide (NiO) Nanopetals for Ultrasensitive Glucose Sensing
Source: Nanoscale Res Lett. 2018 Jan 11;13:16. doi: 10.1186/s11671-018-2435-3 (PMC5764908; doi:10.1186/s11671-018-2435-3)
Supplement: Additional file 1: — Supporting information. (PDF 521 kb) [file 11671_2018_2435_MOESM1_ESM.pdf]

Supporting Information

Mesoporous Nickel Oxide (NiO) Nanopetals for Ultrasensitive Glucose Sensing

Suryakant Mishra, Priyanka Yogi, P.R. Sagdeo and Rajesh Kumar*

Material Research Laboratory, Discipline of Physics & MEMS, Indian Institute of Technology Indore, Simrol-453552 India.

Experimental details

Nickel nitrate precursor mixed with potassium persulfate in the presence of less amount of ammonium solution has been used for the alignment during the preparation of these NiO NSs. After five hours of continuous heating at 150 ^{0}C, deposited film was rinsed with deionized water and dried in air. Subsequently, the NiO-NSs film was annealed at 250 ^{0}C for 2 hours. Uniform and well aligned NiO NSs were obtained on the conducting surface of FTO coated glass. The microstructure of the film was investigated by a XRD (Rigaku SmartLab X-ray diffractometer using monochromatic Cu-Kα radiation λ = 1.54 Å) along with electron microscopy (Supra55 Zeiss). Energy dispersive X-ray spectroscopy (Oxford Instrument) and X-ray Photoelectron Spectrometer (ESCA System, SPECS GmbH, Germany) with Al K_α radiation (1486.6 eV) has been used for the elemental confirmation. Atomic force microscopy has been performed on a Bruker (MultiMode 8-HR) machine and analysis of high resolution nanostructures were carried out using WSxM software.[1] For glucose sensing with NiO-NSs, appropriate electrochemical measurements have been performed using Keithley 2450-EC electrochemical work station. Brunauer–Emmett–Teller (BET) method was also employed on Autosorb iQ, version 1.11 (Quantachrome Instruments) for surface analysis.

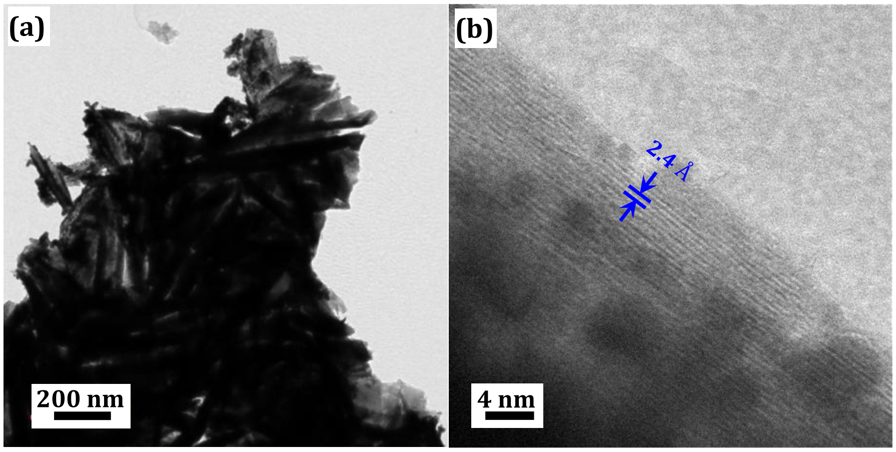

Figure S1: (a) TEM image of the multiple NiO nanopetals (b) HRTEM image of a petal and their corresponding diffraction pattern.

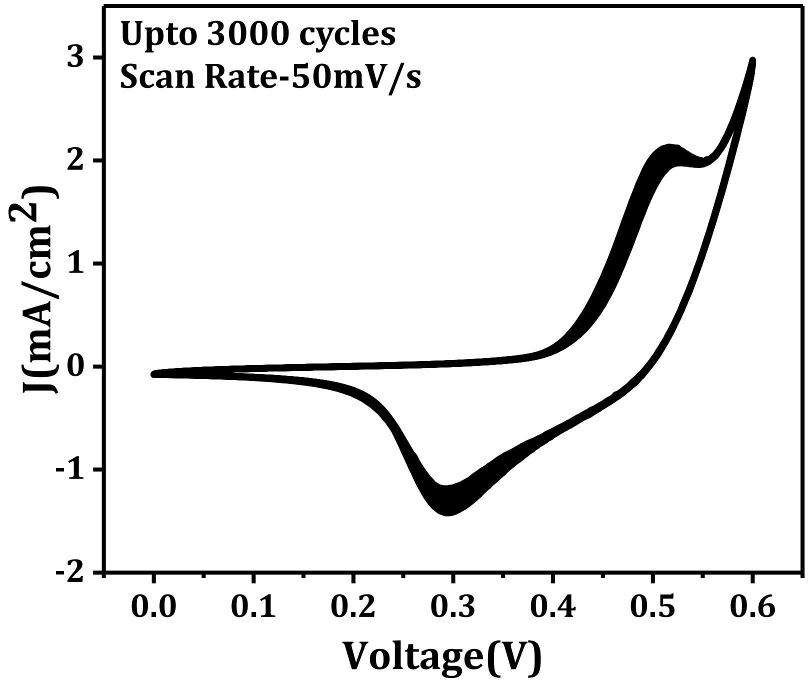

Figure S2: Repeatability test of the fabricated electrode sensor upto 3000 cycles at the scan rate of 50mV/sec

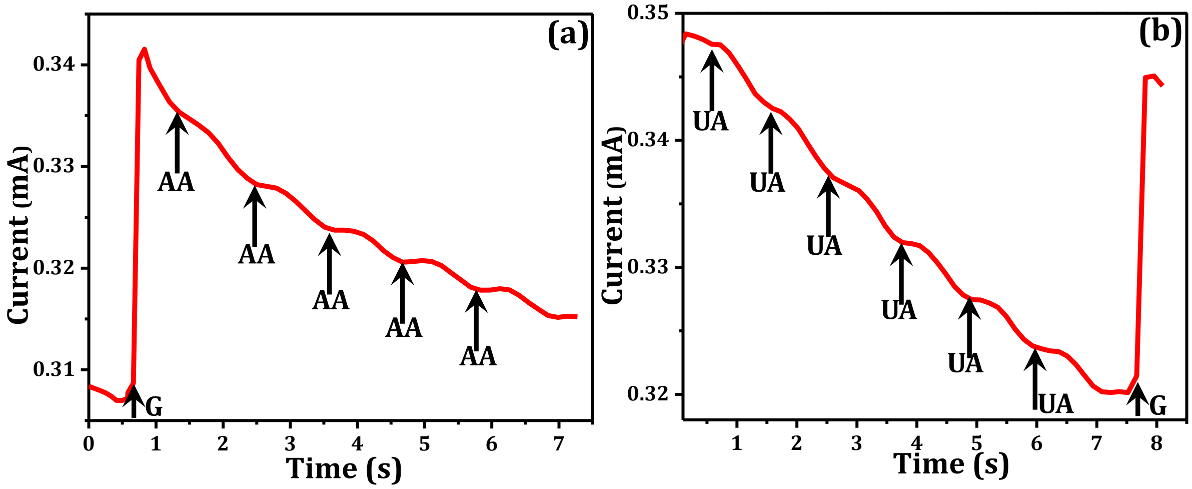

Figure S3: Selectivity measurement of the fabricated electrode using ascorbic acid (AA) and uric acid (UA).
